# Tyndallized bacteria prime bronchial epithelial cells to mount an effective innate immune response against infections

**DOI:** 10.1007/s13577-024-01080-z

**Published:** 2024-05-30

**Authors:** Serena Di Vincenzo, Caterina Di Sano, Claudia D’Anna, Maria Ferraro, Velia Malizia, Andreina Bruno, Marta Cristaldi, Chiara Cipollina, Valentina Lazzara, Paola Pinto, Stefania La Grutta, Elisabetta Pace

**Affiliations:** 1 Institute of Translational Pharmacology (IFT), National Research Council (CNR), Via Ugo La Malfa, 90100 Palermo, Italy; 2 Rimed Foundation, 90100 Palermo, Italy; 3 NBFC, National Biodiversity Future Center, 90100 Palermo, Italy; 4 Dipartimento di Scienze Economiche, Aziendali E Statistiche–Università Degli Studi Di Palermo, 90100 Palermo, Italy; 5 Dipartimento di Sanità Pubblica, Medicina Sperimentale e Forense–Università di Pavia, 27100 Pavia, Italy

**Keywords:** Bronchial epithelial cells, Epithelial homeostasis, Innate immune responses, Tyndallized bacteria

## Abstract

Airway epithelium represents a physical barrier against toxic substances and pathogens but also presents pattern recognition receptors on the epithelial cells that detect pathogens leading to molecule release and sending signals that activate both the innate and adaptive immune responses. Thus, impaired airway epithelial function and poor integrity may increase the recurrence of infections. Probiotic use in respiratory diseases as adjuvant of traditional therapy is increasingly widespread. There is growing interest in the use of non-viable heat-killed bacteria, such as tyndallized bacteria (TB), due to safety concerns and to their immunomodulatory properties. This study explores *in vitro* the effects of a TB blend on the immune activation of airway epithelium. 16HBE bronchial epithelial cells were exposed to different concentrations of TB. Cell viability, TB internalization, TLR2 expression, IL-6, IL-8 and TGF-βl expression/release, E-cadherin expression and wound healing were assessed. We found that TB were tolerated, internalized, increased TLR2, E-cadherin expression, IL-6 release and wound healing but decreased both IL-8 and TGF-βl release. In conclusion, TB activate TLR2 pathway without inducing a relevant pro-inflammatory response and improve barrier function, leading to the concept that TB preserve epithelial homeostasis and could be used as strategy to prevent and to manage respiratory infection, exacerbations included.

**Supplementary Information:**

The online version contains supplementary material available at 10.1007/s13577-024-01080-z.

## Introduction

Multiple factors can contribute to increase respiratory infective risk in humans: (a) altered barrier permeability or increased viral receptors that favor pathogen entry; (b) inadequacy of innate or adaptive immune responses; (c) altered tissue/organ homeostasis due to chronic diseases. The fundamental role of airway epithelium against respiratory infection is now recognized. The perturbation of airway epithelial cell function can profoundly compromise airway integrity and increase the recurrence of airway infections. In fact, the epithelium lining the airway mucosa performs the role of a physical barrier that makes it difficult for pathogens to access [1]. In addition, mainly the proximal than the distal-airway epithelial cells express pattern recognition receptors (PRRs) that promptly sense pathogen presence, release molecules and send signals, activating both innate and adaptive immune responses by specialized immune cells, including antigen presenting cells to amplify innate immune response and to mount appropriate adaptive immune responses against pathogens [1–3]. So, airway epithelial cell functions represent a fundamental bridge between effective innate and adaptive anti-pathogen responses. The recurrence of infections in chronic airway diseases, asthma included, increases the frequency of disease exacerbations that induce in turn disease progression toward the most severe forms of asthma [4, 5]. Therefore, preventing respiratory infections, by controlling anti-pathogen response and by preparing the epithelium in a prompt anti-pathogen reaction is a key factor for disease control. Also, the impairment of the functional activities of natural killer cells (NK) must also be taken into consideration due to the relevant role exerted by NK in the innate immune responses [6, 7]. Bronchial epithelial cells express among PRRs, toll like receptors (TLRs) for gram+ (TLR2) and for gram− (TLR4) bacteria [2, 3, 8]. The increased oxidative stress, as cigarette-smoke exposure, can alter the expression and the activity of TLRs, thus compromising the innate immunity activity exerted by epithelial cells and inducing inflammatory responses that amplify and perpetuate tissue damage [9]. On the other hand, the release of soluble mediators named alarmins as IL-33 and TSLP or of pro-inflammatory cytokines, as IL-8 and IL-6, is consequent to the exposure of airway epithelium to pollutants, microbes, allergens and irritants [10].

Although until very recently lungs were considered sterile, it is now well known that lungs and all the respiratory tract, included nose, oral, upper and lower airways, are colonized by own microbiota, consisting in bacteria, fungi and virus, that modulates both the physiologic functions and the immune surveillance [11]. The pulmonary microbiota is different from gut microbiota, but it is similar in all the respiratory tract and the main bacteria present in healthy adult lungs consists in Firmicutes [12]. Recent evidence highlights the importance of microbiota in the balanced and effective immune responses against external insults [13]. A previous paper assessed that in COPD and in asthmatic patients the bronchial tree contains a characteristic microbiota [14].

The use of heat-killed non-viable probiotics is very interesting as they can be used with greater safety even in vulnerable subjects such as immunocompromised people, neonates and elderly [15]. A technique that allows the bacteria to be inactivated is tyndallization which involves exposing the bacteria to high temperatures (70–100 °C) alternating with incubations at lower temperatures [16]. In this way, the bacteria release components with important immunomodulatory, anti-inflammatory and protective effects on the mucosa [17] [18]. Our recent publication has already demonstrated that the use of a blend of tyndallized bacteria (TB) composed by *Lactobacillus Casei, Lactobacillus Acidophilus, Lactobacillus Plantarum, Streptococcus Thermophilus*, are capable to orientate macrophages polarization toward M1, a functional phenotype that protects from allergic diseases and that can control viral infections thus preventing disease exacerbations [19]. At our knowledge, there is no evidence on the effect of the TB in modulating immune response in bronchial epithelium.

Therefore, the present study is aimed to assess in vitro in the human bronchial epithelial cell line 16HBE the effect of the above cited TB blend on cell viability, cell endocytosis, TLR2 protein and gene expression, IL-6, IL-8 and on TGF-βl gene expression and release, E-cadherin expression and wound healing.

## Material and methods

### Bronchial epithelial cell culture (16HBE)

A human bronchial epithelial cell line immortalized with the origin-of-replication defective SV40 plasmid (pSVori-), 16HBE, were used in this study. 16HBE cells were maintained in a humidified atmosphere of 5% CO_2_ in air at 37 °C and were cultured as adherent monolayers. Eagle’s minimum essential medium (MEM), supplemented with 10% heat-inactivated (56 °C, 30 min) fetal bovine serum (FBS), 1% MEM (non-essential aminoacids), 2 mM L-glutamine and 0.5% gentamicin was used for culturing the cells.

### Cell treatment

16HBE cells were grown to confluence for 24 h and then the culture medium was changed to a medium containing 1% FBS and without antibiotics. Thus, cells were exposed or not to different concentrations (10^6, 5*10^6, 10^7, 5*10^7, 10^8, 5*10^8, 10^9 colony-forming units (CFU)/ml) of a TB blend composed of *Lactobacillus Casei, Lactobacillus Acidophilus, Lactobacillus Plantarum, Streptococcus Thermophilus*. The experiments were repeated at least three independent times and each experiment was performed at least in duplicate.

### Cell viability assay

The effect of TB on cell viability of 16HBE was evaluated as previously reported [20] by MTS assay using the CellTiter 96® Aqueous One Solution Cell Proliferation Assay (Promega, Madison, WI, USA), a colorimetric method capable of determining the number of viable cells by using 3-(4,5-dimethylthiazol-2-yl)-5-(3-carboxymethox-yphenyl)-2-(4-sulfopheyl) 2H-tetrazolium (MTS). 16HBE cells were seeded in 96-well plates and, after 24 h, were treated with the different concentration of TB above reported for 24 h. At the end of the treatment, 20 µL of CellTiter 96® AQueous One Solution reagent was added to each well and the plates were incubated for 20 min at 37 °C and 5% of CO_2_. The absorbance was measured by using a microplate reader at 490 nm (Microplate reader SPECTROstar-Nano, BMG-Labtech, Allmendgrün, Ortenberg). Results were expressed as percentage of viability compared with untreated cells (NT) (100% viability).

### Staining of TB with SytoRed and internalization of TB

The bacterial suspension (at concentration of 10^9 CFU/ml) was incubated with SytoRed Fluorescent Nucleic Acid Stain (20 µM, Molecular Probes, Life Technologies, Thermo Fisher Scientific, MA, USA) for 30 min at room temperature in the dark. The stained suspension of bacteria was washed three times in PBS and then re-suspended in 1 ml of serum- and antibiotic-free medium. 16HBE cells were seeded in 96-well plates and, after 24 h, were incubated for 3 h with 100 µl of medium containing different concentrations of stained bacteria (10^6, 5*10^6, 10^7 CFU/ml). At the end of the incubation, the cells were washed with PBS and fixed with 100 µl 4% paraformaldehyde for 10 min at room temperature. After three washes, cells were stained with DAPI (Abcam, Cambridge, UK) and evaluated by the Operetta CLS system (PerkinElmer, MA, USA) in confocal mode at 63 × magnification.

### Flow-cytometry

16HBE cells were seeded in 6-well plates and, after 24 h, were treated with 1 ml of medium containing different concentrations of SytoRed stained bacteria (10^6, 5*10^6, 10^7 CFU/ml) for 24 h. At the end of stimulation, cells were collected and incubated with a mouse FITC conjugated anti-human TLR2 (eBioscience, San Diego, CA). Negative controls were performed using mouse immunoglobulins negative control (Dako). E-Cadherin expression was also evaluated on 16HBE treated with different concentration of TB (10^6, 5*10^6, 10^7 CFU/ml) for 24 h. At the end of the treatment, cells were collected and incubated with E-cadherin antibody (sc-21791 Santa Cruz Biotechnology, Dallas, Texas, USA) followed by anti-mouse IgG FITC (Dako—Agilent Technologies, Santa Clara, CA, USA). Negative controls were set up using an isotype control antibody (BD PharMingen, Mountain View, CA). For both markers, cells were analyzed by flow-cytometry using a FACSCalibur (Becton Dickinson, Mountain View, CA) for TLR2 and CytoFLEX (Beckman Coulter, Brea, CA, USA) for E-Cadherin expression. Analysis was done on 10,000 acquired events for each sample. Cell debris and dead cells were excluded from the analysis. Data were expressed as percentage of positive cells.

### Western blot analysis

The expression of E-cadherin in total proteins from cell lysates was assessed by western blot analysis as previously described [21]. Protein concentrations were evaluated using the Bradford assay (Biorad, Hercules, CA, USA). 30 μg of lysates were resolved on a 4–15% Mini-PROTEAN TGX Precast Protein Gels, then transferred to nitrocellulose membrane (Biorad, Hercules, CA, USA). The membranes were blocked with Odyssey Blocking Buffer (P/N 927–40,000) and probed with E-Cadherin antibody from Santa Cruz Biotechnology (sc-21791). The membranes were then incubated with IRDye® 800CW Donkey anti-Mouse (LI-COR Biosciences) and protein signal was then detected using the Odyssey® Classic Imaging System (LI-COR) and Image Studio™ Software (LI-COR). For loading control, membranes were re-tested with a mouse anti-β-Actin (Santa Cruz Biotechnology). Contrast and brightness were adjusted equally across entire images to best visualize protein bands.

### Real‐time PCR

16HBE cells were seeded in 6-well plates and, after 24 h, were treated with different concentration of TB (10^6, 5*10^6, 10^7 CFU/ml) for 6 and 24 h. At the end of the treatment, the whole RNA was isolated using TRIzol Reagent (Life Technologies, Thermo Fisher Scientific, MA, USA) following the manufacturer’s instruction. 1 μg of RNA was reverse‐transcribed to cDNA, using iScript cDNA Synthesis kit (Biorad, CA, USA). TLR2, and E-cadherin (CDH-1) expression was evaluated by qRT‐PCR conducted by Step One Plus Real‐time PCR System (Applied Biosystems, Thermo Fisher Scientific, CA, USA) using specific FAM‐labeled probe and primers (prevalidated TaqMan Gene expression assay for TLR2, HS001872448_S1; for CDH-1, Hs001023895_m1; Applied Biosystems) as previously described [22, 23]. Gene expression was normalized to GAPDH (prevalidated TaqMan Gene expression assay for GAPDH, Hs03929097_g1) as endogenous control gene. The relative quantification of mRNA was obtained with the comparative Ct method (2^ − ΔΔCt) and was plotted as respective fold‐change. Untreated cells (NT) were used as reference sample.

### ELISA

16HBE cells were seeded in 6-well plates and, after 24 h, were treated with different concentration of TB (10^6, 5*10^6, 10^7 CFU/ml) for 24 h. At the end of stimulation, the release of IL-6, IL-8, and TGF-β1 protein in cell supernatants was determined as previously reported [20] using human ELISA kit (R&D Systems, MN, USA) following manufacturer’s instructions.

### Wound healing assay

16HBE were seeded in a 6-well plate and were cultured to confluence. Three circular wounds were prepared in each well using a 200-µl pipette tip. After washing with PBS 1X to eliminate debris, cells were allowed to recover for one hour and then incubated with different concentration of TB (10^6, 5*10^6, 10^7 CFU/ml) for 24 h. Digital images were acquired using a digital camera connected to an inverted phase-contrast light microscope at 0 h, 24 h and 48 h after wounding. The surface of the wound area was measured using the ImageJ software to assess remaining wound size and wound closure rates. The results were expressed as percentage of area reduction at time point 24 h (T24) compared to time point 0 h (T0) and at time point 48 h (T48) compared to time point 0 h (T0).

### Statistical analysis

Data were expressed as mean ± SD and analyzed by analysis of variance (ANOVA). A *p* value < 0.05 was considered to be statistically significant. Correlation analyses between the cytokines IL-6, IL-8 and TGF-β1 were evaluated by calculating the parametric Pearson’s correlation coefficient r and a two-tailed *p* value. A *p* value of < 0.05 was considered statistically significant. These analyses and the correlation heatmap have been performed using R software (R, version 4.0.2; R Foundation for Statistical Computing: Vienna, Austria, 2020).

## Results

### Effect of TB on the viability of 16HBE

**Fig. 1:**
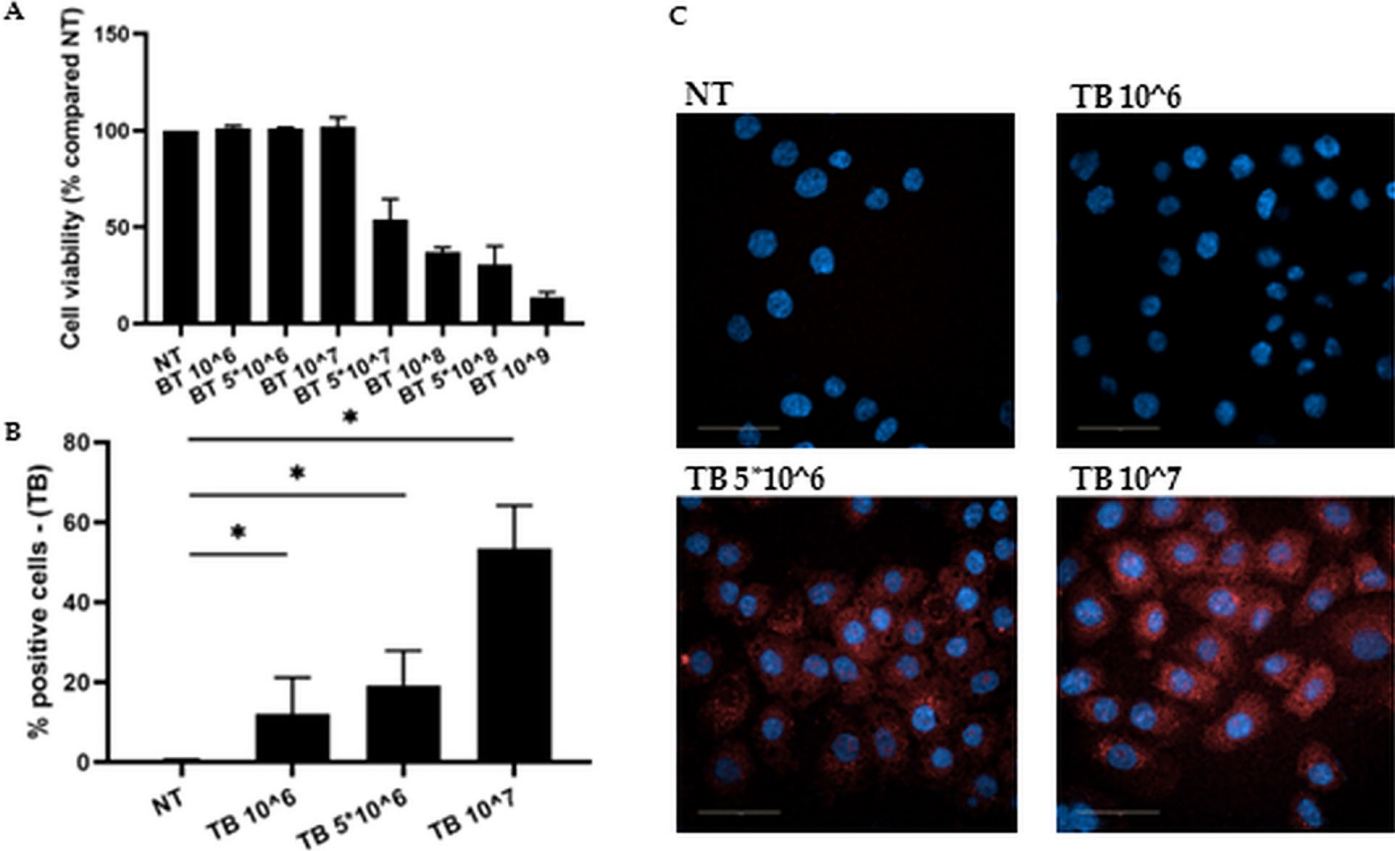
Effect of TB on 16HBE viability and internalization of TB**.** A. 16HBE were incubated with different concentrations of TB for 24 h and the effect on the viability was assessed by MTS assay. Data are expressed as mean ± SD (*N* = 3) of % change compared to NT. * *p* < 0.05 (ANOVA). **B-C**. 16HBE were incubated for 24 h (**B**) and for 3 h (**C**) with different concentrations of Sytored-stained TB and the internalization was evaluated by flow-cytometry (**B**) and by confocal microscopy (C). B. Internalization of the stained bacteria assessed by flow-cytometry. Data are expressed as mean ± SD (*N* = 4) of % positive cells. * *p* < 0.05 (ANOVA). C. Representative images showing internalization of TB (Blue: DAPI, Red: TB)

Cell viability of 16HBE exposed to different concentrations of TB, as above reported, was evaluated by MTS assay. Since TB at 5*10^7, 10^8, 5*10^8, 10^9 CFU/ml significantly decreased cell viability (Fig. 1 A), we therefore used for the subsequent experiments: 10^6, 5*10^6, 10^7.

### Internalization of TB in 16HBE

Since it is well known that endocytosis of bacteria activates bronchial epithelial cells and enforces [24] innate immune responses [25], we tested whether TB were internalized by 16HBE. As shown in Fig. 1 B-C, TB were internalized by 16HBE in a dose-dependent manner.

### Effect of TB on the expression of TLR2 by 16HBE

**Fig. 2:**
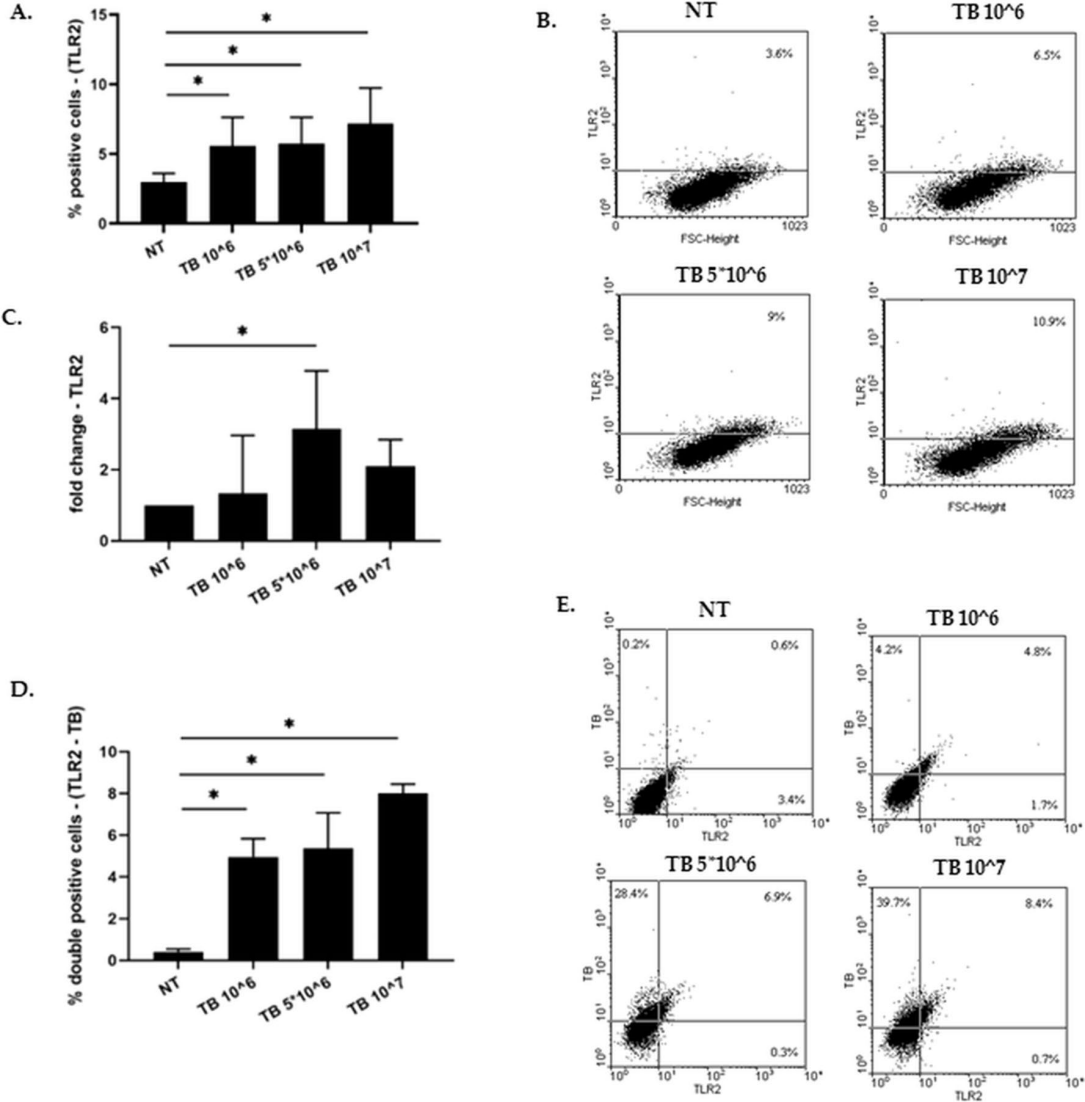
Effect of TB on the expression of TLR2 by 16HBE and correlation between TB internalization and TLR2 expression. **A-C**. 16HBE were incubated for 24 h with different concentrations of TB and the effect on the protein (**A-B**) and gene expression (**C**) of TLR2 were assessed by flow-cytometry and Real Time PCR. Data are expressed as mean ± SD (*N* = 4) of % positive cells and fold change. * *p* < 0.05 (ANOVA). D-E. 16HBE were incubated for 24 h with different concentrations of TB and TLR2 expression and internalization of the stained bacteria by flow-cytometry were evaluated. **D**. Data are expressed as mean ± SD (*N* = 4) of % positive cells. * *p* < 0.05 (ANOVA). **E**. Representative dot-plot from flow cytometric analysis

Since the tested TB blend is composed by Gram+ bacteria [8], TLR2 expression by flow-cytometry was explored. TLR2 expression was significantly increased upon TB incubation in a dose-dependent manner (Fig. 2 A-B). We further investigated whether this effect was due to increased TLR2 gene expression. As shown in Fig. 2C, TB was able to significantly increase also TLR2 gene expression in 16HBE.We therefore evaluated TLR2 expression in correlation to the internalization of the stained bacteria by 16HBE. As shown in Fig. 2 D-E the significant increase of TLR2 expression is related to the internalization of the TB.

### Effect of TB on the gene expression and release of cytokines in 16HBE

**Fig. 3:**
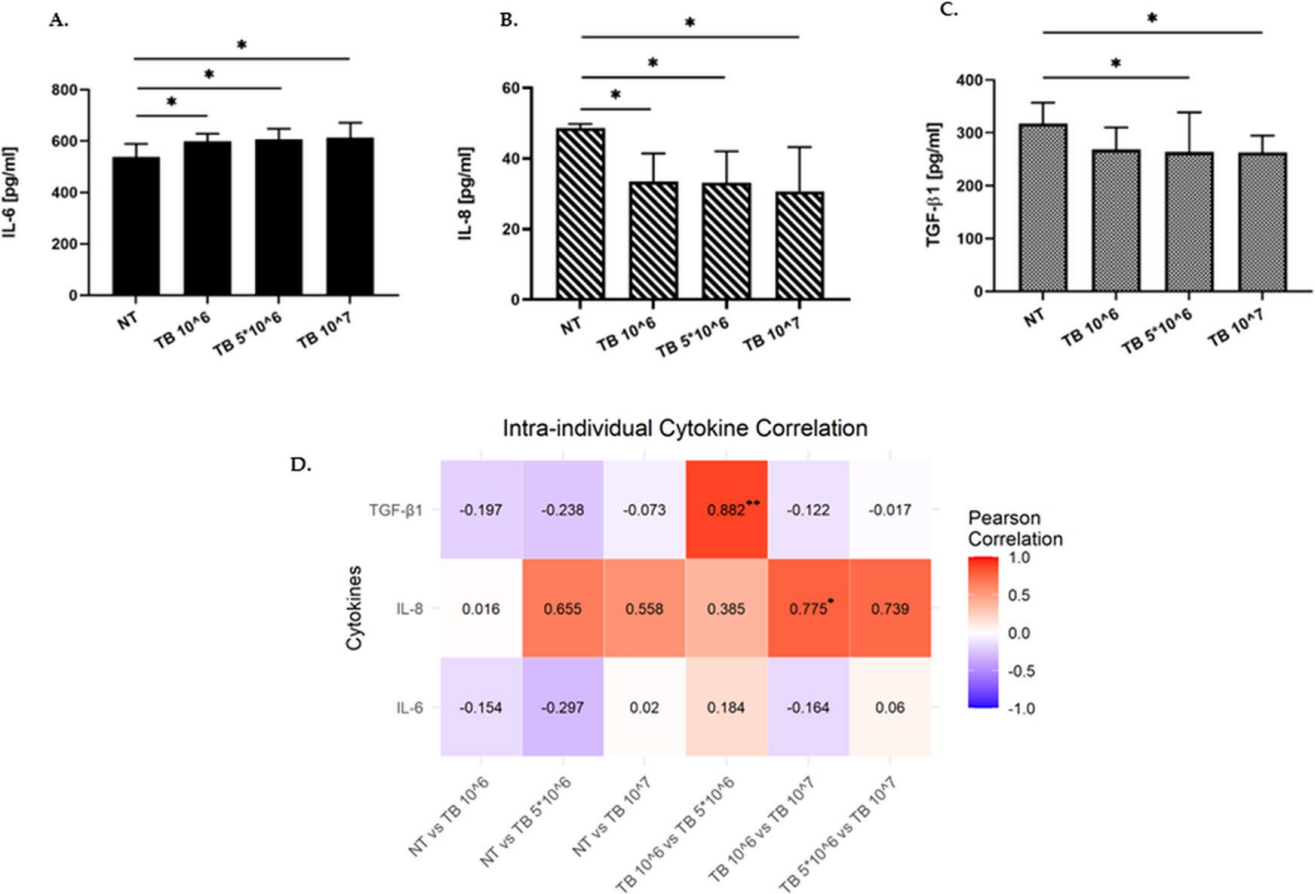
Effect of TB on IL-6, IL-8 and TGF-β1 release by 16HBE**.** A-C.16HBE were incubated for 24 h with different concentrations of TB and the effect on the IL-6 (*N* = 8) (**A**), IL-8 (*N* = 7) (**B**), and TGF-β1 (*N* = 7) (**C**) protein release were assessed by ELISA. Data are expressed as mean ± SD. * *p* < 0.05 (ANOVA). D. Heatmap of intra-individual cytokine Pearson correlation coefficients. Dark red denotes a high positive correlation (*r* → 1), dark blue denotes a high negative correlation (*r* →  − 1), and white denotes a lack of correlation (*r* ≅ 0). ** *p* < 0.01, *p < 0.05 (cor.mtest, R software)

Since activation of TLR2 leads to the release of cytokines, the gene expression and the release of IL-6, IL-8 and TGF-β1 were evaluated. Our results showed that the incubation with TB significantly induced IL-6 release (at all the tested TB concentrations) (Fig. 3A) whereas, significantly decreased IL-8 (at all the tested TB concentrations) and TGF-βl release (at TB 5*10^6 and at TB 10^7) (Fig. 3B, 3C). The analysis of the intra-individual correlations of cytokines among the different experimental conditions showed a significant positive correlation for TGF-β1 between the concentrations TB 10^6 and TB 5*10^6 (*p* value = 0.003) with a Pearson coefficient of 0.882, and between TB 10^6 and TB 10^7 for IL-8 (*p* value = 0.04) with a Pearson coefficient of 0.775 (Fig. 3D). The inter-individual analysis of cytokines did not show any significant correlation (Figure S1).

TB at all the tested concentrations did not affect gene expression of IL-6, IL-8, and TGF-βl after 6 h and after 24 h (Figure S2 A, B, C).

### Effect of TB on the E-cadherin expression in 16HBE

**Fig. 4:**
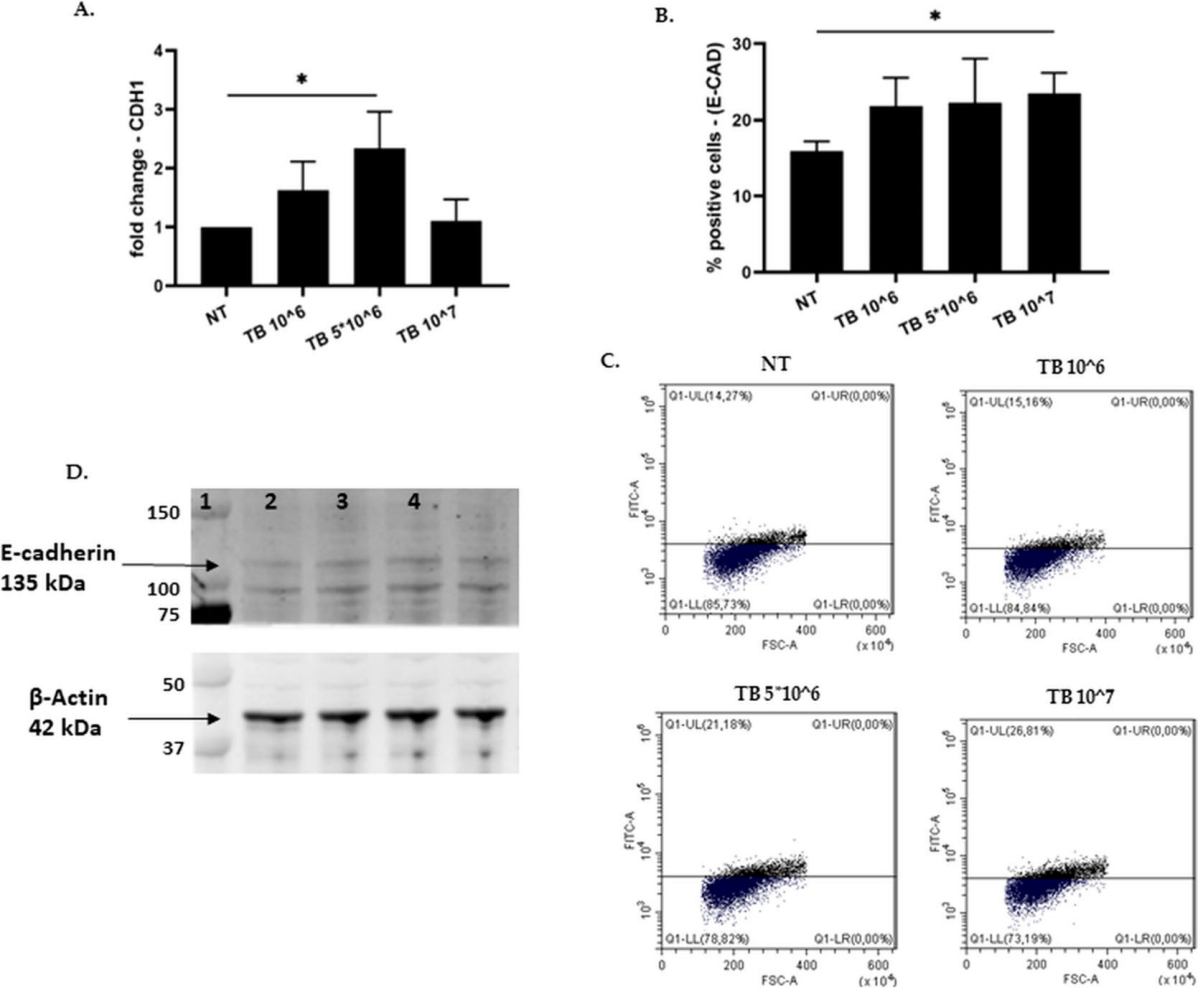
Effect of TB on the gene and protein expression of E-cadherin in 16HBE. 16HBE were incubated for 24 h with different concentrations of TB and the gene (**A**) and protein expression of E-cadherin was assessed by Real Time PCR and by flow-cytometry (**B-C**) or western blot analysis (**D**), respectively. A. Data are expressed as mean ± SD (*N* = 4) of fold change compared to NT. * *p* < 0.05 (ANOVA); B. Data are expressed as mean ± SD (*N* = 4) of % positive cells. * *p* < 0.05 (ANOVA). C. Representative dot-plot from flow cytometric analysis; D. Representative western blot of E-cadherin and β-actin expression (lane 1: NT, lane 2: TB 10^6; lane 3: TB 5*10^6; lane 4: TB 10^7)

E-cadherin mediates adhesion between epithelial cells and plays a fundamental role in preserving mucosal barrier function [26]. We therefore assessed the effect of TB on E-cadherin gene expression. As shown in Fig. 4, TB were able to significantly upregulate E-cadherin gene (at TB 5*10^6) (Fig. 4A) and protein (at TB 10^7) expression (Fig. 4B, C and D).

### Effect of TB on the repair processes in 16HBE

**Fig. 5:**
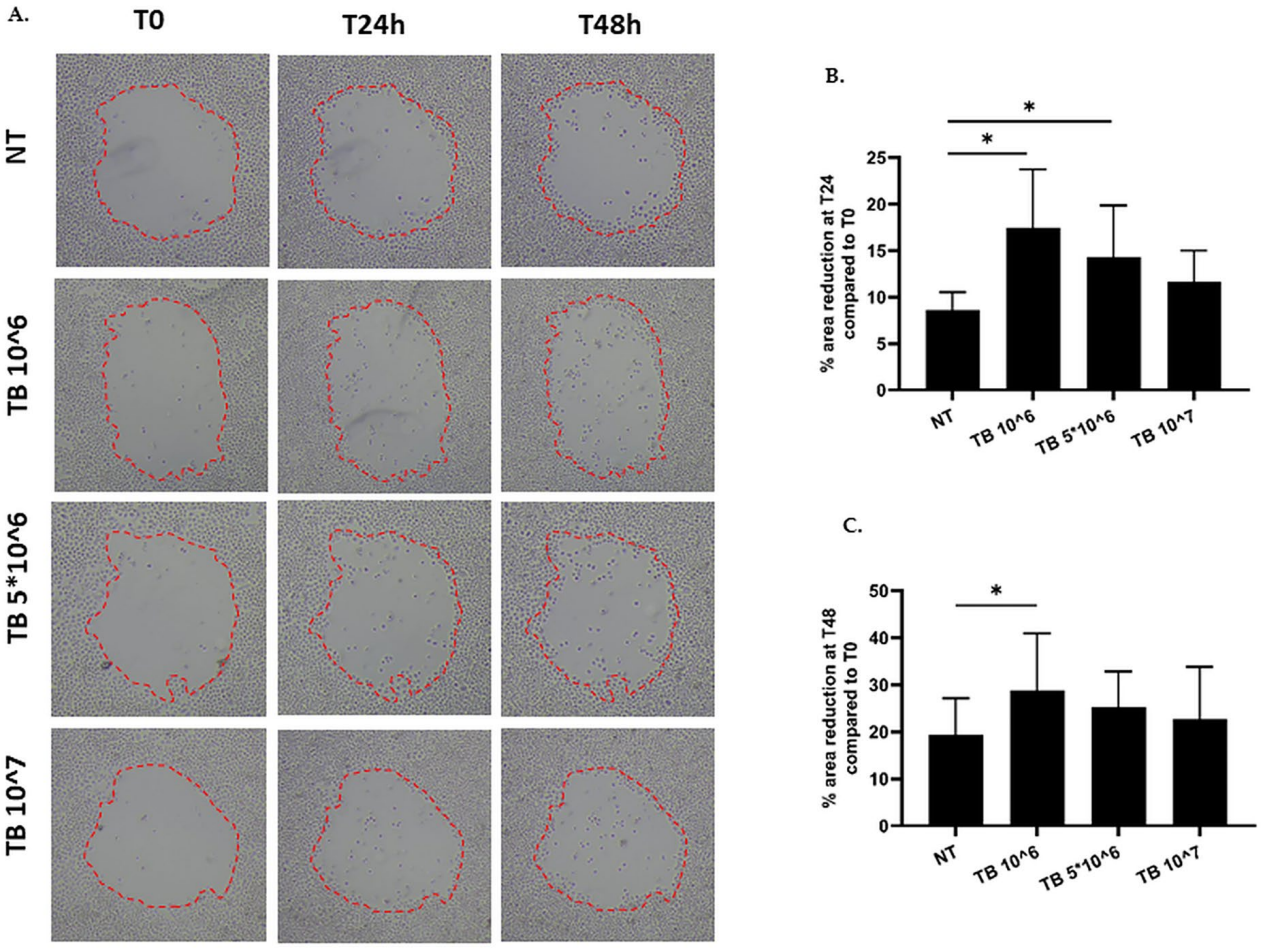
Effect of TB on wound healing. 16HBE were seeded in a 6-well plate and were cultured to confluence. A wound was made in each well using a 200-µl pipette tip. Cells were stimulated with different concentrations of TB for 24 h. **A**. Representative images. **B-C**. The results are expressed as percentage of area reduction at time point 24 (T24) and 48 h (T48) compared to time point 0 h (T0) (*N* = 13) * *p* < 0.05 (ANOVA)

The integrity of epithelial barrier is maintained by effective repair processes in response to injury [27]. Based on this, we explored the effect of TB on repair processes of bronchial epithelial cells by wound healing test. TB (10^6 and 5*10^6 at 24 h and 10^6 at 48 h) significantly improved the repair processes in comparison to untreated cells (Fig. 5 A-C). TB at 10^7 tended to increase both cell proliferation and actin polymerization (data not shown).

## Discussion

The airway epithelium represents the first point of contact for inhaled foreign organisms. The protective arsenal of the airway epithelium is provided in the form of a physical barrier and of a vast array of antimicrobial receptors and compounds that constitute the innate immune system. The airway epithelium also produces several resident antimicrobial proteins, such as lysozyme, lactoferrin, and mucins, as well as a range of cationic proteins. The loss of airway epithelial integrity and recurrence of infective events are relevant mechanisms in promoting allergic and chronic inflammatory airway diseases, such as bronchial asthma. The increased incidence of allergic diseases can be due to alterations in the composition of the airway microbiota due to a reduced exposure to beneficial symbiotic bacteria or parasites [28]. To our knowledge, as of now there are only few pre-clinical studies in sensitized mice for food allergy where the probiotics as *Lactobacillus plantarum JC7* [29], *Lactobacillus rhamnosus 2016SWU.05.0601* [30], and *Lactobacillus Acidophilus, Lactobacillus Delbrueckii subsp, Bulgaricus*, *Lactobacillus Plantarum* can alleviate the allergic symptoms and leading to decrease dysbiosis in gut microbiota, by reducing the production of Th2 cytokines and IgE while increasing TH1 cytokines and T regulatory (Treg) lymphocytes. In the last years, non-pathogenic live microorganisms, also known as probiotics, have been widely used as immunomodulators to improve protection against pathogens and to preserve mucosal barrier integrity [31]. On the other hand, the category of TB represent a set of functional bioactive compounds, derived from microbial fermentation, and included several metabolites, as short-chain fatty acids, as well as microbial cell fractions, functional proteins, and cell lysates. Tyndallized bacteria can have a direct immunomodulatory and clinically relevant effects [32]. Over the last few years, it has been started to use in clinical practice a new category of probiotics, the heat-inactivated probiotics as TB [18, 19] mainly for safety reasons in the most fragile populations, as immunodepressed patients of all ages, neonates and elderly. No data are available on the effects of TB on the bronchial epithelium homeostasis. This has led us to investigate in in vitro model the effect of TB in the 16HBE bronchial epithelial cells, after our previous data with the same TB blend, assessed on macrophage polarization in allergic diseases and infection control [19]. The uptake of commensal and beneficial bacteria by epithelial cells are reported in intestinal epithelial cells and requires endocytic processes that rely on actin microfilament [33]. A previous study (on two bacterial strains of lactobacillus (*BL23* and *LGG*) has documented that these strains are endocytosed by epithelial cells and survive for long periods within these cells without affecting their viability [34]. This tolerability is due to the finding that the mentioned strains induce the secretion of biologically active proteins, P40 and P75, with an anti-apoptotic and proliferative effects linked to activation of EGFR receptor and the Erk/Akt pathway [34]. Here, TB are internalized by bronchial epithelial cells without affecting cell viability as confirmed by MTS results. Many of the known innate immune receptors, including PRRs and TLRs are expressed by the airway epithelium, leading to the production of pro-inflammatory cytokines and chemokines that directly target microorganisms and recruit immune cells, such as neutrophils and T cells, at the site of infection. It is well known that TLR2 activation and signaling, crucial for induction of host immune and defense responses against pathogens, must be tightly controlled. Airway epithelial cells express low levels of TLR2 that can be moved from the apical to basolateral side of the cells and vice versa to ensure effective immune response when pathogens cross the mucosal barrier or to limit tissue damage in absence of benefits for the host [35]. The TLRs responses against commensals should be tightly regulated to maintain tissue homeostasis but TLR2 deficiency predisposes the hosts to bacterial infections, leading to disease exacerbations. It has been previously reported in pre-clinical studies that airway allergic inflammation is characterized by impaired TLR2 expression and reduced IL-6 production that in turn dampens *Mycoplasma* *Pneumoniae* clearance [36]. IL-6 deficiency, in an experimental murine model of asthma, exacerbates disease processes [37], leading to lung inflammation and tissue damage with an increased TGF-β1production. We assessed that TB increases TLR2 and induces the release of IL-6 thus demonstrating that their exposure ameliorates processes that improve innate immune responses against infections. The finding that TB decrease the production of IL-8, confirms that these TB are protective since they do not evoke an unnecessary neutrophil chemotactic gradient that in turn could amply tissue damage. We further explored the effects of TB evaluating TGF-β1 that exerts a relevant role in a process that transforms epithelial cells into mesenchymal cells in a process called epithelial-mesenchymal transition (EMT) [38]. EMT is a protective mechanism for tissue repair but exaggerated and prolonged EMT process can lead to fibrosis and tissue damage [39]. TGF-β1 sharing 42% sequence homology with EGF, can stimulate EGF receptor thus competing with EGF since it colocalises in the same areas of the airways [40]. Taken into account that TGF-β1 is a pro-fibrotic signal, this property derails normal repair processes rather than stimulating them, largely through its promotion of EMT [41]. We found that TB reduces TGF-β1 suggesting that the use of TB strategy prevents a shift from beneficial repair to fibrotic process. Airway remodeling in asthma can be, other than the result of increased activity of TGF-β1, also the result of aberrant and defective repair epithelial processes [27]. To deep inside the beneficial mechanisms promoted by TB exposure, we assessed the effects on barrier function evaluating E-cadherin expression. E-cadherin is one of the main adherent junctions and is a type I transmembrane glycoprotein with an extracellular domain that mediates adhesion between epithelial cells and an intracellular domain linked to cytoskeleton [26]. E-cadherin maintains epithelial cell–cell contacts, regulates cell differentiation and proliferation [42]. Allergens, virus, pollutants and cigarette smoke alter epithelial barrier function reducing E-cadherin expression and function [26]. As consequence of epithelial barrier loss, a relevant release of alarmins including TSLP, IL-33 and IL-25 with innate and adaptive immune response activation occurs. These cytokines promote Type 2 immune responses and IL-4, IL-5, IL-9, IL-13 release with consequent eosinophil accumulation that amplifies tissue damage. Also, TSLP promotes abnormal distribution and cleavage of E-cadherin and in an asthmatic mice model, airway hyperreactivity and airway inflammation that are alleviated by TSLP neutralization [43]. For the first time we demonstrated that an increase in E-cadherin expression is observed upon TB exposure thus confirming a beneficial effect in terms of barrier function. The increased expression of E-cadherin or the reduced release of TGF-β1 (a positive stimulus for activating EMT processes) due to TB exposure, suggests a counteracting effect on EMT processes. In this regard, future studies are needed to clarify whether TB exposure counteract EMT processes in the presence of positive controls including TGF-β1 stimulation.

Lastly, the effect of TB on wound healing was tested. The obtained results document that the repair potential of bronchial epithelial cells was improved by TB exposure. The increased repair potential of airway epithelium upon TB exposure seems to be supported by the associated improvement of both cell proliferation and actin polymerization, an essential mechanism in cell migration.

### Data limitations and perspectives

Since, this is the first experimental study investigating the effect of a blend of TB in bronchial epithelium homeostasis, a limitation of our study is that it is not performed on human primary bronchial epithelial cells nor in animals, but on human bronchial epithelial SV40 immortalized cell line, 16HBE. However, the perspectives are related to the beneficial effects of TB as new preventive and therapeutic tool on proximal airway epithelium in terms of improving barrier function and innate immunity. By improving an innate immune system in the bronchial epithelium, a TB-intervention approach can prevent both the infections and the exacerbations in chronic inflammatory pulmonary diseases as asthma and COPD, in all ages and in all subjects, included who is more susceptible to the impaired immunity. Our results lead the researchers to better investigate on microbiota’s role also in respiratory tract disease, to better identify an effective approach for primary prevention in lung disease progression and for a tailored therapy in lung infections, in a view of sustainability against the abuse of antibiotics when their use is not mandatory. A better understanding of the composition and function of the “healthy” microbiota of the respiratory tract and how dysbiosis interfere in disease progression could be pivotal in the development of new therapeutic strategies, included pre-, pro-, and post-biotics, aimed both at preventing and at restoring lung diseases. TB may represent a change in clinical practice, also representing a new generation of safe and easy standardized products.

### Supplementary Information

Below is the link to the electronic supplementary material.

Supplementary file1 (DOCX 166 KB)

## Data Availability

The data used to support the findings of this study are included within the article.

## Abbreviations

CFU: Colony-forming units
EMT: Epithelial-mesenchymal transition
FBS: Fetal bovine serum
MEM: Eagle’s minimum essential medium
PRRs: Pattern recognition receptors
TB: Tyndallized bacteria
TLRs: Toll like receptors
