# Supplementary material for: Tyndallized bacteria prime bronchial epithelial cells to mount an effective innate immune response against infections
Source: Hum Cell. 2024 May 30;37(4):1080–90. doi: 10.1007/s13577-024-01080-z (PMC11194193; doi:10.1007/s13577-024-01080-z)
Supplement: Supplementary file 1 — Supplementary file1 (DOCX 166 KB) [file 13577_2024_1080_MOESM1_ESM.docx]

**Figure S1. Heatmap of Pearson correlation coefficients among cytokines**

Method S1: Correlation analyses between the cytokines IL-6, IL-8 and TGF-β1 were evaluated by calculating the parametric Pearson’s correlation coefficient r and a two-tailed p value. A p value of <0.05 was considered statistically significant. These analyses and the correlation heatmap have been performed using R software (R, version 4.0.2; R Foundation for Statistical Computing: Vienna, Austria, 2020).

**
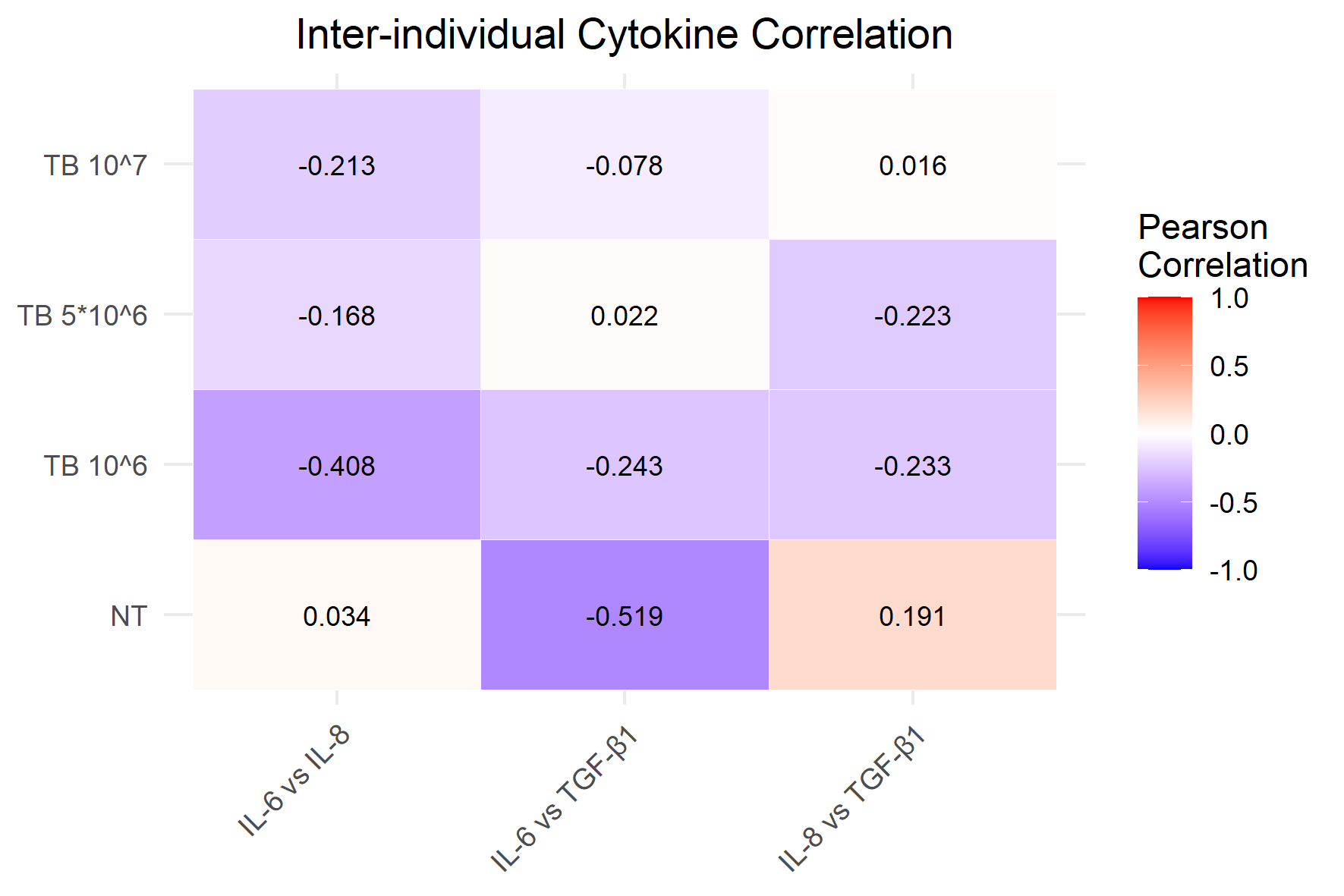
**

Figure S1. Heatmap of Pearson correlation coefficients among cytokines. Dark red denotes a high positive correlation (r → 1), dark blue denotes a high negative correlation (r → −1), and white denotes a lack of correlation (r ≅ 0).

**Figure S2. Effect of TB on cytokine gene expression in 16HBE**

Method S2: 16HBE cells were seeded in 6-well plates and, after 24 h, were treated with different concentration of TB (10^6, 5*10^6, 10^7 CFU/ml) for 6 and 24 h. At the end of the treatment, the whole RNA was isolated using TRIzol Reagent (Life Technologies, Thermo Fisher Scientific, MA, USA) following the manufacturer’s instruction. 1 μg of RNA was reverse‐transcribed to cDNA, using iScript cDNA Synthesis kit (Biorad, CA, USA). IL-6, IL-8 and TGF-β1 expression was evaluated by qRT‐PCR conducted by Step One Plus Real‐time PCR System (Applied Biosystems, Thermo Fisher Scientific, CA, USA) using specific FAM‐labeled probe and primers (prevalidated TaqMan Gene expression assay for IL-8, Hs00174103_m1; for IL-6, Hs00985639_m1; for TGF-β1, Hs00998133_m1 Applied Biosystems). Gene expression was normalized to GAPDH (prevalidated TaqMan Gene expression assay for GAPDH, Hs03929097_g1) as endogenous control gene. The relative quantification of mRNA was obtained with the comparative Ct method (2^−ΔΔCt) and was plotted as respective fold‐change. Untreated cells (NT) were used as reference sample.


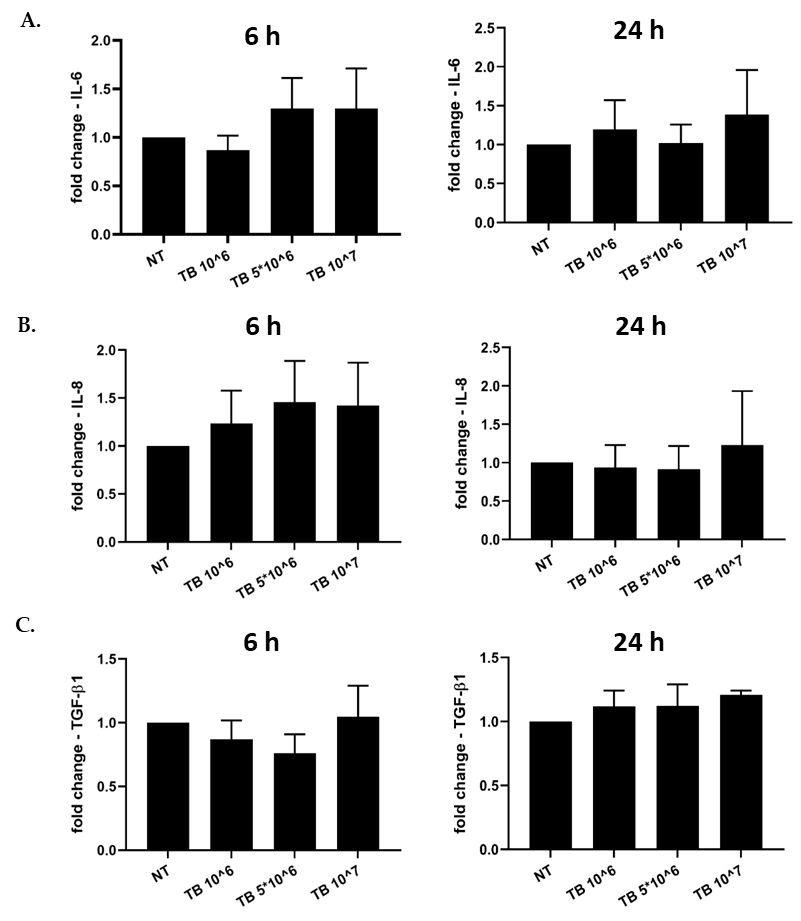


Figure S2. Effect of TB on IL-6 (A), IL-8 (B), and TGF-β1 (C) gene expression in 16HBE. 16HBE were incubated for 6 and 24 h with different concentrations of TB and the gene expression was assessed by Real Time PCR. Data are expressed as fold change.
